# Epigenetic Alterations in Muscular Disorders

**DOI:** 10.1155/2012/256892

**Published:** 2012-06-18

**Authors:** Chiara Lanzuolo

**Affiliations:** CNR Institute of Cellular Biology and Neurobiology, IRCCS Santa Lucia Foundation, Via Del Fosso di Fiorano 64, 00143 Rome, Italy

## Abstract

Epigenetic mechanisms, acting via chromatin organization, fix in time and space different transcriptional programs and contribute to the quality, stability, and heritability of cell-specific transcription programs. In the last years, great advances have been made in our understanding of mechanisms by which this occurs in normal subjects. However, only a small part of the complete picture has been revealed. Abnormal gene expression patterns are often implicated in the development of different diseases, and thus epigenetic studies from patients promise to fill an important lack of knowledge, deciphering aberrant molecular mechanisms at the basis of pathogenesis and diseases progression. The identification of epigenetic modifications that could be used as targets for therapeutic interventions could be particularly timely in the light of pharmacologically reversion of pathological perturbations, avoiding changes in DNA sequences. Here I discuss the available information on epigenetic mechanisms that, altered in neuromuscular disorders, could contribute to the progression of the disease.

## 1. Introduction

Although every cell within our body bears the same genetic information, only a small subset of genes is transcribed in a given cell at a given time. The distinct gene expression of genetically identical cells is responsible for cell phenotype and depends on the epigenome, which involve all structural levels of chromosome organization from DNA methylation and histone modifications up to nuclear compartmentalization of chromatin [1–5]. Enormous progress over the last few years in the field of epigenetic regulation indicated that the primary, monodimensional structure of genetic information is insufficient for a complete understanding of how the networking among regulatory regions actually works. The contribution of additional coding levels hidden in the three-dimensional structure of the chromosome and nuclear structures appears to be a fundamental aspect for the control of the quality and stability of genetic programs. Damage or perturbation of epigenetic components may lead to deviations from a determined cellular program, resulting in severe developmental disorders and tumour progression [6, 7]. Moreover, for human complex diseases, the phenotypic differences and the severity of the disease observed among patients could be attributable to inter-individual epigenomic variation. Unravelling the intricacies of the epigenome will be a complex process due to the enormity and dynamic nature of the epigenomic landscape but is essential to gain insights into the aetiology of complex diseases. 

## 2. The Complexity of the Epigenome

The epigenome consists of multiple mechanisms of transcriptional regulation that establish distinct layers of genome organization and includes covalent modification of DNA and histones, packaging of DNA around nucleosomes, higher-order chromatin interactions, and nuclear positioning [4]. The first layer of epigenetic control is the DNA methylation, an heritable epigenetic mark typically associated with a repressed chromatin state [8], which seems to play a role, together with other histone modifications, in preventing gene reactivation [9]. Vertebrate genomes are predominantly methylated at cytosine of the dinucleotide sequence CpG (for a review see [3]). Despite the high level of CpG methylation, some regions of mammalian genomes are refractory to this modification [10]. These regions, called CpG islands, contain high levels of CpG dinucleotides [11] and localize at or near gene promoters [12], suggesting a strong correlation between differential methylation of CpG islands and flanking promoter activity. From the mechanistic point of view, DNA methylation can inhibit gene expression by blocking the access of transcriptional activators to their binding site on DNA or by recruiting chromatin modifying activities to DNA (for a review see [3]). For long time, DNA methylation was considered as a stable epigenetic mark. However, recently it has been shown that methylated cytosines could be converted to 5-hydroxymethylcytosines (5hmeC) by Tet (Ten eleven Translocation) family proteins [13–15] and the generation of 5hmeC is a necessary intermediate step preceding active demethylation of DNA [16]. The second level of epigenetic regulation occurs through posttranslational histone modification. Histone proteins assemble into a complex that associates with DNA forming the elementary unit of chromatin packaging: the nucleosome. The amino and carboxy termini of the histones (histone tails), protruding from the nucleosome, play an essential role in controlling gene expression, being the target for posttranscriptional modifications, including acetylation, methylation, phosphorylation, ubiquitylation, biotinylation, and several others (for a review see [2, 17]). Multiple histone modifications can also coexist on the same tail, dictating specific biological readouts [18–24]. In addition to histone modifications, a fraction of chromatin contains one or more variant isoforms of the canonical histones that can be incorporated into specific regions of the genome throughout the cell cycle and are essential for the epigenetic control of gene expression and other cellular responses (for a review see [25]). Combinatorial histone modifications and variants play an important role in folding nucleosomal arrays into higher-order chromatin structures, creating local structural and functional diversity and delimiting chromatin subdomains then subjected to a specific protein environment. Chromatin higher-order structures established at DNA level give signals that are recognized by specific binding proteins that in turn influence gene expression and other chromatin functions [1, 26]. This represents an additional layer of epigenetic gene regulation and includes factors, such as transcriptional repressors or activators, that recognizing specific chromatin patterns regulate the folding or modulate the activity of RNA Polymerase II (Pol II). 

The topological organization of chromatin and the association of regulatory elements with specific components of the eukaryotic nucleus is another parameter to be considered in the complexity of the epigenetic information. It is now clear that specific chromosomal conformations, mediated by cis-trans interactions, are associated with distinct transcriptional states in many organisms, allowing the establishment of chromatin boundaries between promoters and regulatory element (for a review see [27, 28]). The nuclear localization also influences gene expression, regulating its access to specific machinery responsible for specific functions, such as transcription or replication [29, 30]. In addition, due to its highly dynamic nature, the genome moves in the nucleus driving specific genomic regions toward nuclear compartments defined by a high concentration of specific factors and substrates that facilitate more efficient biological reactions [31]. This constant motion plays also a role in coordinating the expression of coregulated genes, separated by longer chromosomal regions or located on different chromosomes [32].

The evolutionarily conserved Polycomb group of proteins (PcG) are multiprotein complexes that play a central role during development [1]. The most characterized PcG-encoded protein complexes are Polycomb Repressive Complex 1 (PRC1) and 2 (PRC2). Three other complexes were characterized in *Drosophila*, PHO-repressive complex (PhoRC), dRing-associated factors (dRAF) complex, and Polycomb repressive deubiquitinase (PR-DUB), and their components have orthologues in mammals [33, 34]. PcG complexes mediate gene silencing by regulating different levels of chromatin structures. Biochemical studies revealed that Enhancer of zeste 2 (EZH2), the Histone Methyl Transferase (HMTase) subunit of PRC2, marks lysine 27 of histone H3 [35–38] and PRC1 complex monoubiquitylates Lys 119 of histone H2A [39]. Moreover, the H3K27me3 mark constitutes a docking site for the chromodomain present in PRC1 components [35], determining a sequential recruitment of PRC complexes, although recent chromatin profiling studies evidenced that PRC1 and PRC2 also have targets independent of each other [40, 41]. Examination of the localization of PcG proteins in the nucleus has revealed that they are organized into distinct domains called Polycomb or PcG bodies, which are often localized, closed to pericentric heterochromatin [42]. PcG targets are frequently localized in PcG bodies in the tissue where they are repressed, suggesting that such nuclear localization may be required for efficient silencing [43, 44]. However, the number of PcG bodies is less than the number of PcG target genes, implying that several PcG targets share the same body. FISH studies together with Chromosome Conformation Capture (3C) analysis have confirmed this coassociation [43–46] and revealed that PcG-dependent higher-order structures organization is conserved in mammals [47–49]. The characteristic feature of the PcG memory system is inheritability of gene expression patterns throughout the cell cycle, ensured by the PcG capability to bind its own methylation mark [50, 51] and specific cell cycle-dependent dynamics [52–55]. Besides their extensively described role in development, in the last years emerging evidence has shown PcG involvement in several other biological processes, such as X chromosome inactivation, differentiation, and reprogramming (reviewed in [56–58]). The highly variability of PcG functions and the fine quantitative and qualitative tuning of their activities is generated by the association of different PcG proteins and their coregulators in a combinatorial fashion and/or by the regulation of their recruitment at specific chromatin sites (reviewed in [1, 59, 60]). One recent example is a genomewide study of TET complex localization, in murine Embryonic Stem (ES) cells. This complex, responsible for 5hmeC generation, colocalizes with a subpopulation of Polycomb-repressed genes, contributing to gene transcription control [61–63].

## 3. Muscle Diseases

Skeletal muscles are composed by multiple aligned multinucleated cells, the myofibers, wrapped in a plasma membrane called sarcolemma. Inside the sarcolemma and all around the myofibers, there is a specialized cytoplasm, the sarcoplasm, that contains the usual subcellular elements [64]. A plethora of structural molecules and cellular proteins connecting all the fibers components together with specialized signalling pathways and transcription factors are required for a correct muscle formation and function. Dysfunction or lack of any component of the skeletal muscle could lead to a muscular disorder, the muscular dystrophy (MD), clinically characterized by muscle weakness and skeletal muscle degeneration [64]. Some dystrophies arise from mutations of molecules that play a role outside the nucleus while other dystrophies derive from dysfunction of the nucleus or its membrane. The nonnuclear dystrophies include Duchenne MD (DMD), Becker MD (BMD), and all MD affecting proteins working in the sarcoplasm. DMD is the most severe form of muscular dystrophy and is caused by mutations that preclude the production of the essential cytoskeletal muscle protein dystrophin, which anchors proteins from the internal cytoskeleton to a complex of proteins (dystrophin-associated protein complex, DAPC) on the membrane of muscle fibers [65]. This interaction is important for the structural stabilization of the sarcolemma [66]. Interestingly, recent reports highlighted the influence of epigenetic mechanisms regulating histone deacetylation (HDAC) pathways in the development of this disease [67–69] and the reversion of some DMD-associated phenotypes in presence of inhibitors of HDACs [70, 71]. 

The nuclear dystrophies include all MD generated by a dysfunction of nuclear membrane (laminopathies) or by expansion or contraction of nucleotide repeats, not necessarily contained in a coding region, which affect nuclear function. Myotonic dystrophy is the most common MD in adult and is a complex multisystemic inherited muscle degenerative disorder caused by a pathogenic expansion of microsatellite repeats within noncoding elements of dystrophia myotonica protein kinase (*DMPK*) or zinc finger protein 9 (*ZNF9*) genes [72]. These expansions, although transcribed into RNA, do not affect the protein-coding region of any other gene. However, it has been shown that transcripts accumulate in the nucleus and interfere with protein families that regulate alternative splicing during development [64, 73]. In this paper, I will describe the contribution of epigenetic mechanisms mediated by Polycomb group of proteins to two human nuclear muscular dystrophies, facioscapulohumeral muscular dystrophy (FSHD), and laminopathies.

## 4. Polycomb Group of Protein as Epigenetic ****Regulators of Muscle Differentiation

PcG proteins regulate large numbers of target genes, primarily those involved in differentiation and development [74–80]. During cell differentiation the progressive restriction of the developmental potential and increased structural and functional specialization of cells ensure the formation of tissues and organs [57]. Myogenesis is a multistep process that starts with the commitment of multipotent mesodermal precursor cells. Upon appropriate stimuli these cells differentiate and fuse into multinucleated myotubes, giving rise to the myofibers. In mammals, PcG proteins are primarily involved in muscle differentiation by binding and repressing muscle-specific gene regulatory regions in undifferentiated myoblasts to prevent premature transcription. During myogenesis progression, PcG binding and H3K27me3 are lost at muscle-specific loci, resulting in appropriate muscle gene expression [81–84]. Interestingly, artificial modulation of EZH2 levels, either by depletion or overexpression, consistently affects normal muscle differentiation, accelerating or delaying, respectively, muscle cell fate determination [82, 83, 85]. Although emerging evidence suggested a key role for epigenetic mechanisms in muscular diseases [68, 71, 86–89], the precise contribution of Polycomb proteins to the pathology and progression remains largely unexplored.

## 5. Facioscapulohumeral Muscular Dystrophy**** (FSHD)

Facioscapulohumeral muscular dystrophy (FSHD) is a frequent (1 : 15.000) dominant autosomal miopathy that is characterized by progressive, often asymmetric weakness and wasting of facial (facio), shoulder, and upper arm (scapulohumeral) muscles [90]. Monozygotic twins with different penetrance of FSHD have been described, suggesting a strong epigenetic contribution to the pathology [91, 92]. Genetically, FSHD1, one of the two forms of FSHD, is caused by a contraction of the highly polymorphic D4Z4 macrosatellite repeat in chromosome 4q [93]. In the general population, this repeat array varies between 11 and 100 units of 3,3 kb each, ordered head to tail [94]. Most patients with FSHD1 present a partial deletion of the D4Z4 array, which leaves 1–10 units on the affected allele [93]. Although a linear negative correlation between repeat size and clinical severity has not been observed, some findings indicated that smaller D4Z4 arrays result in earlier disease onset and enhanced severity in patients [95–97]. Interestingly, at least one D4Z4 unit is necessary to develop FSHD, as monosomy of 4q does not cause the disease [98]. In addition to polymorphism associated with D4Z4 repeat number, two allelic variants of the 4q subtelomere, termed 4qA and 4qB, have been identified. These variants differ for the presence of a *β* satellite repeat immediately distal to the D4Z4 array on 4qA allele [99]. Whereas 4qA and 4qB chromosomes are almost equally common in the population, FSHD arises mainly from 4qA haplotype [99–102]. D4Z4 repeat arrays are not restricted to chromosome 4q, but homologous sequences have been identified on many chromosomes [103]. In particular, the subtelomere of chromosome 10q is almost identical to the region in 4q containing D4Z4 repeats, containing highly homologous and equally polymorphic repeat arrays [104, 105]. However, chromosome 10 with less than 11 repeat units does not cause FSHD1 [106], suggesting that the chromatin environment associated with chromosome 4q and/or 4q-specific DNA sequences could contribute to FSHD development. In agreement with this observation, the relatively gene-poor region flanking D4Z4 repeats on chromosome 4q contains two attractive candidates that have been characterized for their contribution to disease development: FRG1 (FSHD Region Gene 1) and the double-homeobox transcription factor DUX4. FRG1 is highly conserved in both vertebrates and invertebrates and it has been found overexpressed in some FSHD samples [107, 108]. Moreover, transgenic mice overexpressing FRG1 develop, selectively in the skeletal muscle, pathologies with physiological, histological, ultrastructural, and molecular features that mimic human FSHD [109]. However, FRG1 overexpression in FSHD samples is not a uniform finding [110, 111] and thus the contribution of the FRG1 gene to the FSHD phenotype needs further validation. Although some evidence suggests a role for FRG1 in pre-mRNA splicing [109, 112, 113], to date the mechanism of action and the role of FRG1 in FSHD onset and development is largely unknown. 

Aberrant production of DUX4, the gene present in the D4Z4 array, was detected in both FSHD1 and FSHD2 muscle biopses [114], suggesting that D4Z4 could affect disease progression [115]. However, D4Z4 array has a complex transcriptional profile that includes sense and antisense transcripts and RNA processing [116]. The DUX4 mRNA is generated by transcription of the last, most distal, unit of the array, including a region named pLAM, which contains a polyadenylation signal, necessary for DUX4 transcript stabilization [115]. The absence of this polyadenylation signal on chromosome 10 suggests its involvement in FSHD development [100]. The DUX4 pre-mRNA can be alternatively spliced [116] and there has been found a DUX4 mRNA isoform encoding for the full-length protein, expressed in FSHD muscle, whereas healthy subjects present an alternative splicing mRNA encoding for a truncated protein [114]. 

DUX4 RNA and protein levels have been arguments of debate in the field for several years. Previous works demonstrated a proapoptotic function for DUX4 [117] and DUX4 overexpression was found to have dramatically toxic effect on cell growth [118]. On the other hand extremely low levels of DUX4 were found in FSHD muscles raising some doubts on the role of this gene in FSHD development [114, 119]. In a recent report, overexpression of DUX4 mRNA in human primary myoblasts followed by gene expression analysis showed deregulation of several genes involved in RNA splicing and processing, immune response pathways, and gametogenesis [119]. These genes were found aberrantly expressed in both FSHD1 and FSHD2 muscles while a partial recovery of the repressed state occurs upon depletion of endogenous DUX4 mRNA. Although no direct evidence was presented about the role of deregulated genes in the FSHD development, these findings suggest that critical DUX4 protein and RNA levels could be responsible for gene transcription deregulation in FSHD [119]. 

Aside putative genes involved in the FSHD development there is a general consensus in the field in supporting the view that epigenetic mechanisms are important players in FSHD, affecting the severity of the disease, its rate in progression, and the distribution of muscle weakness [120, 121]. Increasing evidence suggested that, in patients, chromatin conformation of FSHD locus is altered at multiple levels, from DNA methylation up to higher-order chromosome structures, resulting in perturbation of heterochromatic gene silencing in the subtelomeric domain of the long arm of chromosome 4. As stated previously, DNA methylation is associated with gene silencing and defects in methylation are generally associated with deregulation of transcriptional programs and disease [6]. D4Z4 is overall very GC-rich, having characteristics of CpG islands [122], and in healthy subjects is methylated, while contracted D4Z4 is always associated with an hypomethylation [123, 124]. Interestingly, FSHD2 patients, which phenotypically show FSHD though lacking D4Z4 contractions, display general D4Z4 hypomethylation [123], indicating an important epigenetic condition necessary to develop or generate the disease.

Combination of posttranslation histone modifications establishes a specific code that recruits nuclear factors responsible of several functions such as transcriptional or replication control. The D4Z4 repeat array is enriched of two repressive marks: trimethylation of lysine 9 or 27 of histone H3 (H3K9me3 and H3K27me3, resp.). The first, generally associated with constitutive heterochromatin, is deposited by the histone methyltransferase SUV39 and is responsible for HP1 repressor recruitment [125]. H3K27me3 is characteristic of facultative heterochromatin, is deposited by the PRC2 subunit EZH2, and in turn recruits PRC1 and PRC2 to establish transcriptionally repressed domains. It has been shown that H3K27me3 and the two Polycomb proteins YY1 and EZH2 are bound to D4Z4 and FRG1 promoter in myoblasts [107, 108] and are reduced during myogenic differentiation [108]. Interestingly, DNA association studies, by using 3C technologies [126], revealed that D4Z4 physically interacts with FRG1 promoter and this DNA loop is reduced upon differentiation. These epigenetic signatures dynamics during myogenesis are accompanied by a gradual upregulation of FRG1 [108]. Conversely, in FSHD1 myoblasts the D4Z4-FRG1 promoter interaction is reduced and FRG1 expression is anticipated during differentiation, suggesting an alteration of epigenetic signatures dynamics occurring when the differentiation starts. Notably, H3K27me3 can still be detected by ChIP at D4Z4 repeats in FSHD1 myoblasts, although by 3D immuno-FISH it was found specifically reduced on D4Z4 on 4q chromosome in FSHD1 myoblasts compared with controls [108]. This apparent inconsistency is justified by the extensive duplication of D4Z4 sequences in the human genome and the limitation of ChIP assay to distinguish specific 4q D4Z4 repeat. In addition to the complex heterochromatic features found at D4Z4 locus, there has been shown the presence of histone marks associated with transcriptional activation in the first proximal D4Z4 unit of the array, such as acetylation of histone H4 and di-methylation of Lys 4 of histone H3 [110, 125]. This could reflect the complexity of bidirectional transcriptional activity at the locus and could suggest the potential presence of noncoding RNA that further regulate the transcription. 

As stated before, epigenetic chromatin regulation depends also on appropriate intranuclear positioning. Most nuclear events do not occur randomly in the nucleoplasm, rather regulatory proteins are spatially clustered in specific territories, and the position of chromosomal region in the nucleus influences its transcriptional activity. The 4q subtelomere is preferentially localized in the nuclear periphery in both controls and FSHD patients [127, 128], and this localization is evolutionary conserved [129]. In FSHD1 cells, this localization depends on a sequence within D4Z4 unit that tethers the subtelomere in the nuclear periphery in a CTCF and Lamin-A-dependent manner [130]. Although intranuclear positioning of 4q subtelomere does not change during muscle differentiation, when several epigenetic modifications take place [108], it has been shown that the nuclear periphery localization in controls and FSHD1 cells can be directed by different sequences, proximal or within D4Z4 repeat, respectively. This suggests that the nuclear environment of FSHD locus in normal or affected subjects could be different and could contribute to the disease development [130].

In summary, the epigenetic analysis suggests that probably the presence of more than ten D4Z4 repeats provides a physiological heterochromatization and repression of the subtelomeric region, due to the saturating levels of epigenetic repressors. In this view, less than ten D4Z4 repeats could be considered as *border line* genotype, because the correct heterochromatin formation is not ensured, determining a predisposition to the disease and also explaining the high variability in disease severity even in the same genetic background. This hypothesis is reinforced by the evidence that patients with less than 3 repeats have more chances to develop FSHD1 disease and that asymptomatic carriers of D4Z4 deletion are increasingly evident in FSHD [131]. Another complex issue about FSHD is the requirement for at least one D4Z4 repeat for the development of the disease, suggesting a *gain of function* effect, where the presence of an aberrant transcription of coding or noncoding RNA or dysregulated binding of epigenetic factors recruited by the D4Z4 array could be necessary for disease development. Systematic analysis of epigenetic modifications across the entire genome in FSHD1 and FSHD2 patients will be crucial to dissect epigenetic mechanisms acting specifically on D4Z4 locus and involved in FSHD pathogenesis and progression.

## 6. Laminopathies

Thenuclear scaffold (or nuclear matrix)is the network of fibersfound inside a cell nucleus. The lamina is the major component of nuclear matrix and is constituted by a complex meshwork of proteins closely associated with the inner nuclear membrane [132]. In vertebrates, lamins have been divided into A and B types, based on sequence homologies. All A-type lamins, A, C, C2, and Δ10, are encoded by alternative splicing of a single gene (LMNA) while two major mammalian B-type lamins, B1 and B2, are encoded by different genes (LMNB1 and LMNB2) [133]. All major lamins terminate with a CAAX-box that is involved in numerous posttranslational modifications including the farnesylation of the cysteine, removal of the-AAX, and carboxymethylation of the cysteine [134]. These modifications are thought to be important for the efficient targeting of the lamins to the inner nuclear membrane [135]. Moreover, Lamin A is further processed by the zinc metalloproteinase, Zmpste24/FACE1, which catalyzes the removal of additional 15 residues from Lamin A C-terminus including the farnesylated and carboxymethylated cysteine [136]. Expression of the A- and B-type lamins is developmentally regulated in mammals, resulting in cell type-specific complements of lamins [137]. In the last years, genome wide studies describing lamin bound chromosomal regions were focalized specifically on B-type [138, 139]. However, it is becoming increasingly evident that A-type lamins are scaffolds for proteins that regulate DNA synthesis, responses to DNA damage, chromatin organization, gene transcription, cell cycle progression, cell differentiation, cancer invasiveness, and epigenetic regulation of chromatin [140–143]. In line with this observation, lamin distribution in the nucleus is type specific, with Lamin B being predominantly present at inner nuclear membrane and Lamin A also present in lower concentrations, throughout the nucleoplasm [144], suggesting, for the latter, a role beyond the maintenance of mechanical stability of the nucleus. Genetic studies confirmed this hypothesis, showing that A- or B-type lamin mutations have different impacts on organisms. Mutations in genes encoding B-type lamins are not frequently connected to diseases in human and Lamin B1 null mice die during early postnatal life with severe defects in their lung and bones [145] while mice lacking Lamin B2 die shortly after birth with severe brain abnormalities. Taken together, these findings indicate that B-type lamins play a structural role in the nucleus essential for cell and tissue function. On the other hand, mice lacking A-type lamins have apparently normal embryonic development [146], but postnatal growth is delayed and they develop abnormalities of cardiac and skeletal muscle. This is in line with studies in human, where a large number of mutations of Lamin A/C (LMNA) were found, causing a wide range of human disorders, including lipodystrophy, neuropathies, autosomal dominant Emery-Dreifuss muscular dystrophy (EDMD), and progeria. The latter includes Hutchinson-Gilford progeria syndrome (HGPS), atypical Werner syndrome, restrictive dermopathy, and mandibuloacral dysplasia type A (MADA) [147]. Collectively, these degenerative disorders with a wide spectrum of clinical phenotypes are known as the laminopathies. To date, despite the identification of several mutations on Lamin A causing these disorders, it is difficult to correlate phenotype to genotype in laminopathies. It is still unclear how specific mutations result in a particular tissue-specific laminopathy phenotype [148] or why a single mutation in Lamin A gene can result in different phenotypes [149]. This suggests an involvement of the individual epigenetic background to the disease. Studies in HGPS cells confirmed this hypothesis finding several epigenetic alterations. In particular there has been shown a decrease of the heterochromatin mark H3K9me3 in pericentric regions and a downregulation of the PRC2 component EZH2, accompanied by a loss of H3K27me3 on the inactive X chromosome (Xi), which leads to some decondensation of the Xi [150]. Notably, it is not clear if observed epigenetic defects are cause or consequence of the irreversible cascade of cellular mechanisms dysfunction accompanying HGPS progression. Most inherited LMNA mutations in humans cause disorders that selectively affect striated muscle, determining decreased levels of A-type lamins. This was confirmed in the Lmna null mice, which develop abnormalities of cardiac and skeletal muscle reminiscent of those seen in human subjects [146]. Remarkably, in humans, decreased lamin A levels observed in some laminopathies could also be dependent on dominant negative effect caused by an aberrant form of Lamin A. Indeed, overexpression in transgenic mice of a human lamin A variant responsible for Emery-Dreifuss muscular dystrophy determines severe heart damage [151]. Several hypotheses have been proposed to explain molecular mechanisms underlying muscular dystrophies caused by lamin A mutations. The current model suggests that the prolonged exposure to mechanical stress of muscle cells determines the tissue-specific degeneration observed in laminopathies. This model takes into consideration only the structural role of lamin A, neglecting its functional role in chromatin organization and gene expression control. There is a growing body of evidence indicating that several signalling pathways, such as pRb, MyoD, Wnt-*β* catenin, and TGF-*β*, are altered in laminopathies [152, 153]. The Rb-MyoD-crosstalk is one of the most described pathways altered in laminopathies. MyoD is a master transcription factor of muscle differentiation that activates muscle-specific genes. Its levels are modulated by dephosphorilated pRb, which takes part in the acetylation and expression of MyoD [154]. Lamin A controls Rb levels favouring its dephosphorilation [155]. Thus in the absence of Lamin A the level of hypophosphorilated Rb and consequently the level of MyoD are reduced, determining a defect in muscle cells' differentiation [156]. This was confirmed by a decreased number of MyoD positive nuclei observed in skeletal muscle from laminopathy patients [157]. Given its role in muscle-specific genes regulation, PcG protein could be involved in aberrant gene expression observed in lamin A defective background. Several indirect evidences support this hypothesis indicating a potential crosstalk between PcG proteins and Lamin A. As mentioned previously the nuclear positioning of the PcG-regulated FSHD locus, responsible for the described neuromuscular disorder, is altered in human Lamin A/C null cells [127]. However, while the role of PcG proteins in governing local chromatin higher-order structures was extensively addressed [44, 47, 49], it is still unknown if they also control the chromosomal position in the nucleus and if the peripheral localization of FSHD is dependent on PcG proteins. Recently, it has been suggested that nuclear position of PcG proteins could be crucial for muscle differentiation [158]. In this work, Wang and colleagues have shown that the localization of PRC2 complex at the nuclear periphery is mediated by the myogenic regulator, Msx1, and is required for a correct repression of Msx1 target genes. This localization occurs in myoblasts and is necessary for a proper muscle differentiation [158]. The importance of chromatin architecture dynamics during muscle differentiation was further confirmed by studies performed by Mattout et al. in *C. elegans* [159]. Using ablation of the unique lamin gene in worm they found that lamin is necessary for perinuclear positioning of heterochromatin. Then, to test the physiological relevance of this association in developing animals, they monitored tissue-specific changes in nuclear position of specific genomic regions in worms that express a dominant mutant form of lamin, which mimics the human Emery-Dreifuss muscular dystrophy. They found that in lamin defective background, muscle-specific genes are not able to relocalize from the nuclear periphery to a more internal location and this determines loss of muscle integrity. Although there has been extensively shown the crucial role of Polycomb proteins in mediating nuclear chromatin architecture, to date no evidence supports a direct involvement of PcG in muscle genes relocalization during normal differentiation. Further studies are needed to determine if physiological epigenetic dynamics that ensure a correct myogenesis are altered in lamin defective background and the role of Polycomb proteins in this process.

## 7. Conclusions

In the last years the study of the epigenome and its role in human disease progression has attracted considerable interest. The insurgence of epigenetic deregulation in human pathologies suggests that specific diseases might benefit from epigenetic-targeted therapies and this type of drug therapy is becoming a reality in clinical settings [160, 161]. Notably, epigenetic variation could arise as a consequence of the disease. Distinguishing epigenetic variations causing or contributing to the disease process is not straightforward but is nevertheless crucial to elucidate the functional role of the disease-associated epigenetic variation and to optimize their utility in terms of diagnostics or therapeutics. Recent advances in genomic technologies, by the expanding use of next-generation DNA sequencing (ChIP-seq) to assess the genomic distribution of histone modifications, histone variants, DNA methylation, and epigenetic factors, will be helpful to study human disease-associated epigenetic variation at genomewide level. Combined with appropriate statistical and bioinformatic tools [162], these methods will give us a more complete picture of all the loci that are epigenetically altered, although they will not resolve the *cause or consequence* issue. Then, the functional characterization of the variety of epigenetic modifications at specific loci could provide insight into the function of these modifications in normal development and in subsequent transition to disease states. These studies could ultimately lead to the future development of more effective epigenetic-based therapies, although treatment with these classes of drugs should be carefully examined to determine whether the therapeutic benefits outweigh the potential adverse effects [163, 164].
